# Supplementary material for: Optimization of Printed Polyaniline Composites for Gas Sensing Applications
Source: Sensors (Basel). 2022 Jul 19;22(14):5379. doi: 10.3390/s22145379 (PMC9319473; doi:10.3390/s22145379)
Supplement: Supplementary file 1 [file sensors-22-05379-s001.zip › sensors-1794522-supplementary.pdf]

# Supporting Information

## S1) Sensor circuit principle

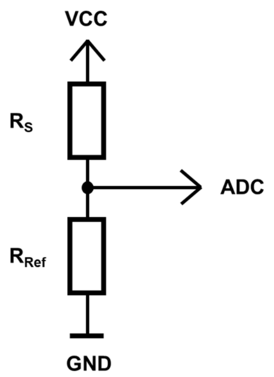


**Figure S1:** Schematic of a sensor unit, consisting of measurement and reference electrode depicted as resistors $R_s$ and $R_{Ref}$, respectively. The midpoint voltage between $R_s$ and $R_{Ref}$ is converted via analog-to-digital converter (ADC) for data logging and further processing on a PC.

## S2) Calculations of the liquid phase to gas phase transfer of analytes

During the experiments, the liquid inlet flow rate of the LCU was set to 2.5 µL/min, the gas flow to 500 SCCM. The concentrations of the prepared dilutions were 449 mM, 223 mM, 90 mM, 45 mM, 9 mM, 4 mM, 1 mM. At the provided liquid and gas flow settings, the selected liquid phase concentrations resulted in gas phase concentrations of 50, 25, 10, 5, 1, 0.5 and 0.01 ppm respectively. The numbers were obtained by evaluation with the calculation tables shown in Figure S5.

First, the densities, molecular weights and respective volumes of both analyte *compound A* (E.g., ammonia hydroxide) and liquid carrier *medium B* (E.g., distilled water) are used to calculate the liquid ppm in a mixed standard (Figure SX) of *compound A* being dissolved in *medium B*. The liquid parts per million of this mixed standard are the molar fraction of compound A over medium B times a million (Equation 1).

$$ppm_{Liquid\ (Mixed\ Standard)} = \frac{\left(\frac{\rho * V}{MW}\right)_{Compound\ A}}{\left(\frac{\rho * V}{MW}\right)_{Medium\ B}} * 1{,}000{,}000 \quad \text{(S1)}$$

Second, the desired liquid flow rate set within the LCU from the liquid inlet to the nozzle of the evaporation chamber is converted from µl/minute ($\dot{V}$) to mol/minute ($\dot{n}$) (Equation 2), using the molarity ($M_{Medium\ B}$) of the liquid carrier medium B, and then, using the ideal gas volume constant $R$, further to standard cubic centimeters per minute (SCCM) (Equation 3). Together with the gas flow provided by an external gas source in SCCM and the previously calculated ppm in the liquid mixed standard, the liquid flow in SCCM is then used to calculate the ppm within the air flow starting at the nozzle of the evaporation chamber (Equation 4):

$$\dot{V} * M_{Medium\,B} = molar\ flow\ \dot{n} \tag{S2}$$

$$\dot{n} * R = SCCM_{Liquid} \tag{S3}$$

$$\frac{ppm_{Liquid} * SCCM_{Liquid}}{SCCM_{Gas} + SCCM_{Liquid}} = ppm_{Gas} \tag{S4}$$

**Table S1**: Calculation table for analyte concentrations in gas phase resulting from liquid phase solutions as used in the liquid calibration unit, provided by IONICON. (part 1)

| | | | |
|---|---|---|---|
| **Calculation of LCU concentration** | | | |
| **Preparing/mixing the liquid standard (first dilution step)** | | | |
| | **Select your VOC** | | **Ammonia hydroxide (28-30%)** |
| dilute | 90.0 | µl | Amount of Ammonia hydroxide (28-30%) |
| | 0.090 | ml | Amount of Ammonia hydroxide (28-30%) |
| | **0.900** | g/ml | Density of Ammonia hydroxide (28-30%) |
| | **35.05** | g/mol | Molecular weight Ammonia hydroxide (28-30%) |
| | **Miscible** | | Solubility |
| | 0.081 | g | Total weight Ammonia hydroxide (28-30%) |
| | 2.31E-03 | mol | Total mol Ammonia hydroxide (28-30%) |
| | **Diluted in solvent** | | **Water** |
| in | 10 | ml | Amount of Water |
| | 998 | kg/m^3 | Density of Water 20°C |
| | 0.998 | g/ml | Density of Water 20°C |
| | 18.00 | g/mol | Molecular weight Water |
| | 0.055 | mol/ml | |
| | 9.98 | g | Total weight Water |
| | 5.5E-01 | mol | Total mol Water |
| | **Mixed Standard** | | |
| results in | 4,167.13 | ppm (liquid) | particle mixing ratio, liquid |
| | 4.2E-03 | mol/mol | mixing ratio, liquid |
| | 0.997 | g/ml | approximate density |
| **Evaporation in LCU** | | | |
| | **Liquid Flow** | | |
| set | 2.5 | µl/minute | FC liq |
| | 0.002500 | ml/min | FC liq |
| | 1.39E-04 | mol/min | FC liq |
| | 1.24 | sccm | µl liquid --> ml-gas |
| | 3.11 | sccm | H2O as gas flow |
| | **Gas flows - normal** | | |
| set | 500 | sccm | FC dil (air) |
| | **Resulting concentration of Ammonia hydroxide (28-30%) in the gas phase** | | |
| the conc. is | 28,581.39 | ppb | of Ammonia hydroxide (28-30%) |
| | 28.5814 | ppm | |
| | 0.62% | absolute humidity | |
| **Constans and References** | | | |
| | **Constants** | | |
| | 22.4 | l/mol | Ideal gas volume |
| | 22400 | ml/mol | Ideal gas volume |

## S3) Sensor response evaluation

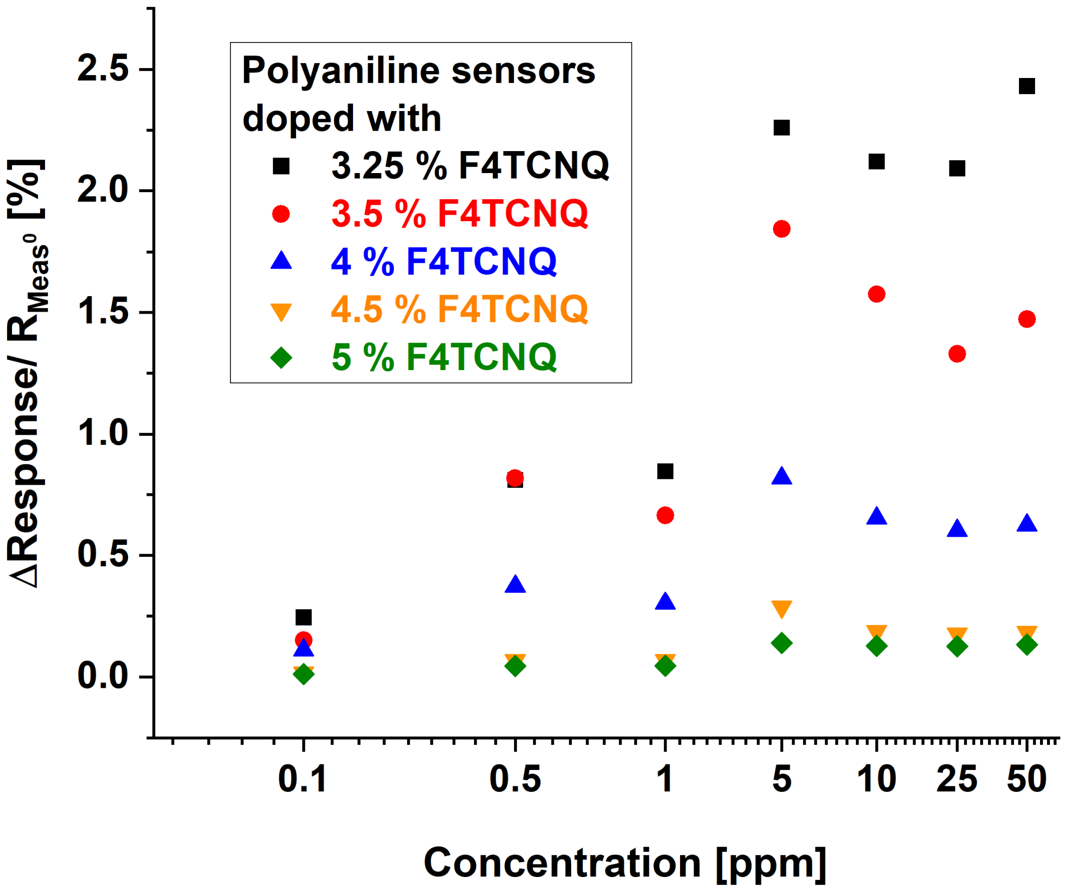


**Figure S2:** Responses of the units shown in the manuscript Figure 3 after 20 minutes of analyte injection.

## S4) Detailed time resolution of Figure 2 in the main manuscript.

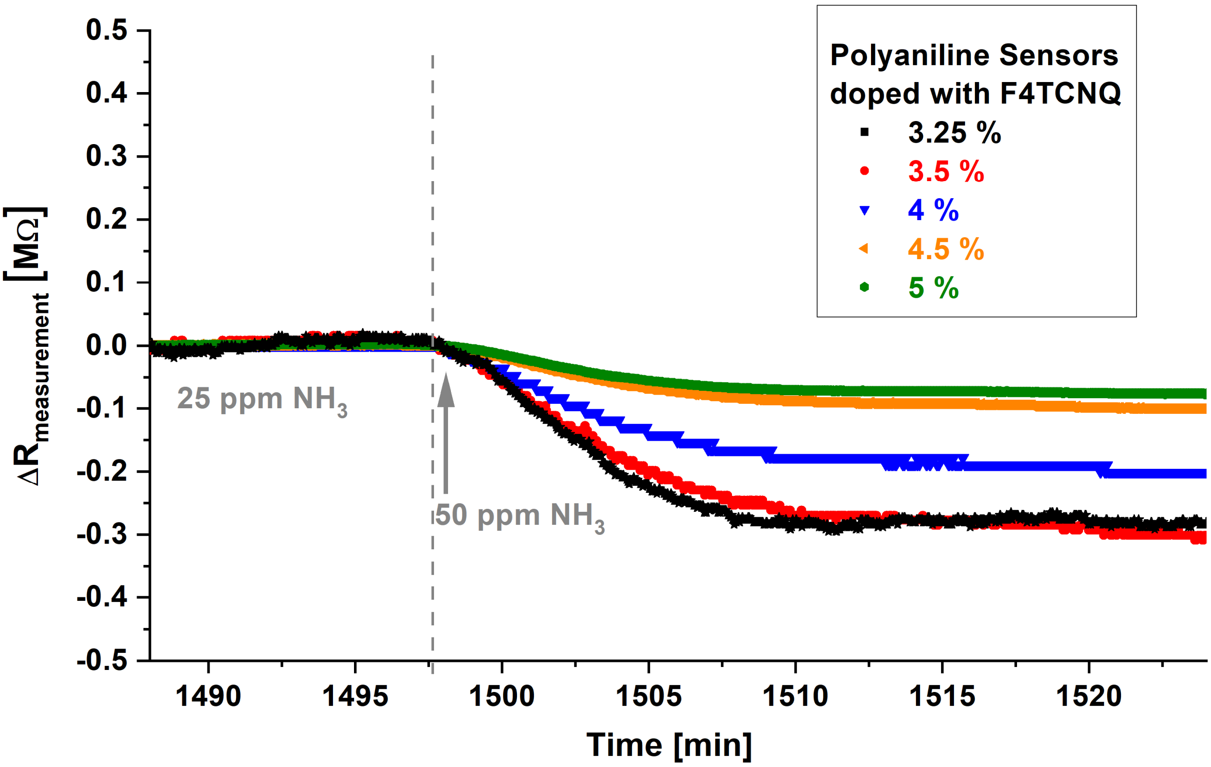


**Figure S3:** Detailed kinetic view of the 50 ppm ammonia injection from the measurement shown in the manuscript Figure 2. For clarity the baselines were normalized so that under the equilibrium conditions of the 25 ppm response all sensor signals are set to zero.

## S5) Titration experiments for demonstration of sensor baseline drift for high analyte concentrations

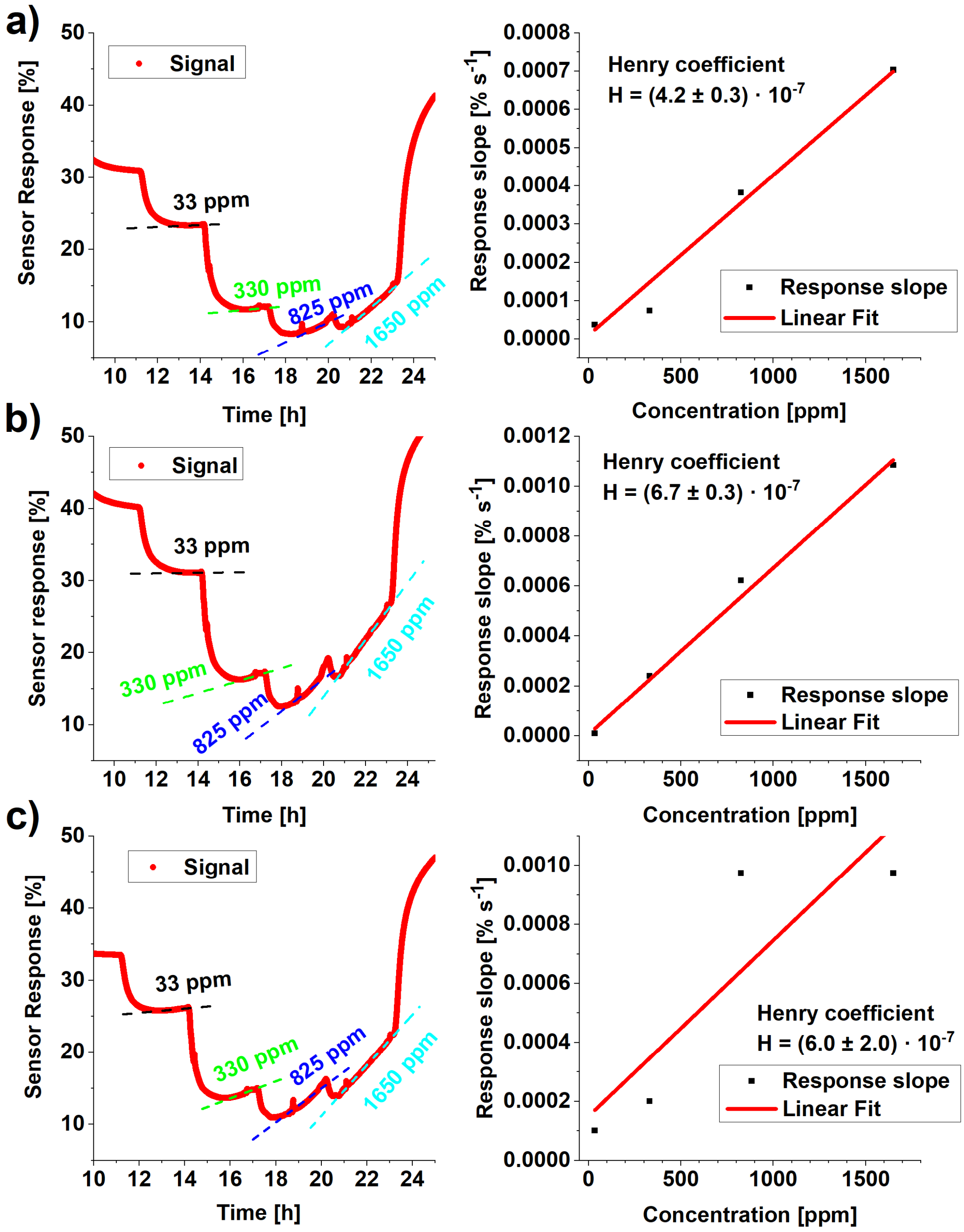


**Figure S4:** Kinetic evaluation of the baseline drift dependence on the injected analyte concentration. Here titrations of ammonia up to 500 ppm are shown for three different sensors. The coefficient of the sensor response slope with the analyte concentration was named "Henry coefficient". (a) a sensor unit with a resulting Henry coefficient of $(4.2 \pm 0.3) \cdot 10^{-7}$ %/ppm s and a fitting R2 of 0.984; (b) a sensor unit with a resulting Henry coefficient of $(6.7 \pm 0.3) \cdot 10^{-7}$ %/ppm s and a fitting R2 of 0.993; (c) a sensor unit with a resulting Henry coefficient of $(6.0 \pm 2.0) \cdot 10^{-7}$ %/ppm s and a fitting R2 of 0.722.

## S6) Calculations of the ppm inside the pre-sampling system for the 6 analytes

The vapor pressures at 20°C for ammonium hydroxide, hexane, acetonitrile, butan-2,3-dione, ethanol and propan-2-ol are 288 kPa, 16 kPa, 9.7 kPa, 7.6 kPa, 7.9 kPa and 6 kPa, respectively. The evaporation rates of the chemicals can then be calculated according to the procedure proposed by Mackay and Wesenbeeck [45] and is approximately 2.5 µg $cm^{-2}$ $h^{-1}$ for ammonium hydroxide. As 3 ppm of ammonia in a volume of 1.7 litre correspond to a total mass of 2.25 µg ammonia in the gas phase this means that at this rate the ammonia hydroxide solution must have evaporated for approximately one hour to achieve 3 ppm. This is in very good agreement with the applied sample timing of 6 analytes for 5 minutes with 5 minutes of regeneration time, meaning that the box for ammonia was indeed accumulating for 60 minutes. Applying the same procedure for all analytes one yields the following evaporation rates shown in Table S2. The surface area of the paper stripe and the liquid surface is calculated to approximately 1 $cm^2$ and the evaporation time of the chemicals before opening of the lids after sampling was 1 hour. Therefore, the concentrations in the sampling chamber calculated in this manner are shown in the last column of Table 1.

| Chemical | MW (g $mol^{-1}$) | Evaporation rate (µg $cm^{-2}$ $h^{-1}$) | Sampling concentration (ppm) |
|---|---|---|---|
| Ammonium hydroxide | 17.03 | 2.25 | 5 |
| Acetonitrile | 41.05 | 6 | 5.6 |
| Ethanol | 46.07 | 6.7 | 5.6 |
| Hexane | 86.18 | 12.6 | 5.6 |
| Butane-2,3-dione | 86.09 | 12.6 | 5.6 |
| Propan-2-ol | 60.1 | 8.7 | 5.6 |

**Table S2:** Calculated sample concentrations in the headspace sampling system after exposure accumulation of gaseous compounds for the timeframe of the measurements shown in Figure 4.

In summary, the signal transduction to the sensor surface depends highly on the adsorption affinity and the diffusion parameters which are highly specific for each molecule.

### Average sensor responses to the 6 analytes:

| Chemical | Average and standard deviation for the sensor responses of Polyaniline + 4.25 wt% F4TCNQ | Average and standard deviation for the sensor responses of Pristine Polyaniline |
|---|---|---|
| Ammonium hydroxide | - 4.4 ± 1.0 % | - 2.7 ± 0.6 % |
| Acetonitrile | - 2.3 ± 0.5 % | - 1.9 ± 0.4 % |
| Ethanol | -1.0 ± 0.3 % | - 0.3 ± 0.5 % |
| Hexane | - 0.4 ± 0.2 % | - 0.4 ± 0.1 % |
| Butane-2,3-dione | + 1.3 ± 0.6 % | + 1.9 ± 0.6 % |
| Propan-2-ol | - 0.3 ± 0.2 % | - 0.2 ± 0.2 % |

**Table S3:** Statistical analysis of the responses and standard deviations of pristine and F4TCNQ doped sensors as shown in the measurement shown in Figure 4.

## S7) Kinetics of the sensor responses to six different analytes

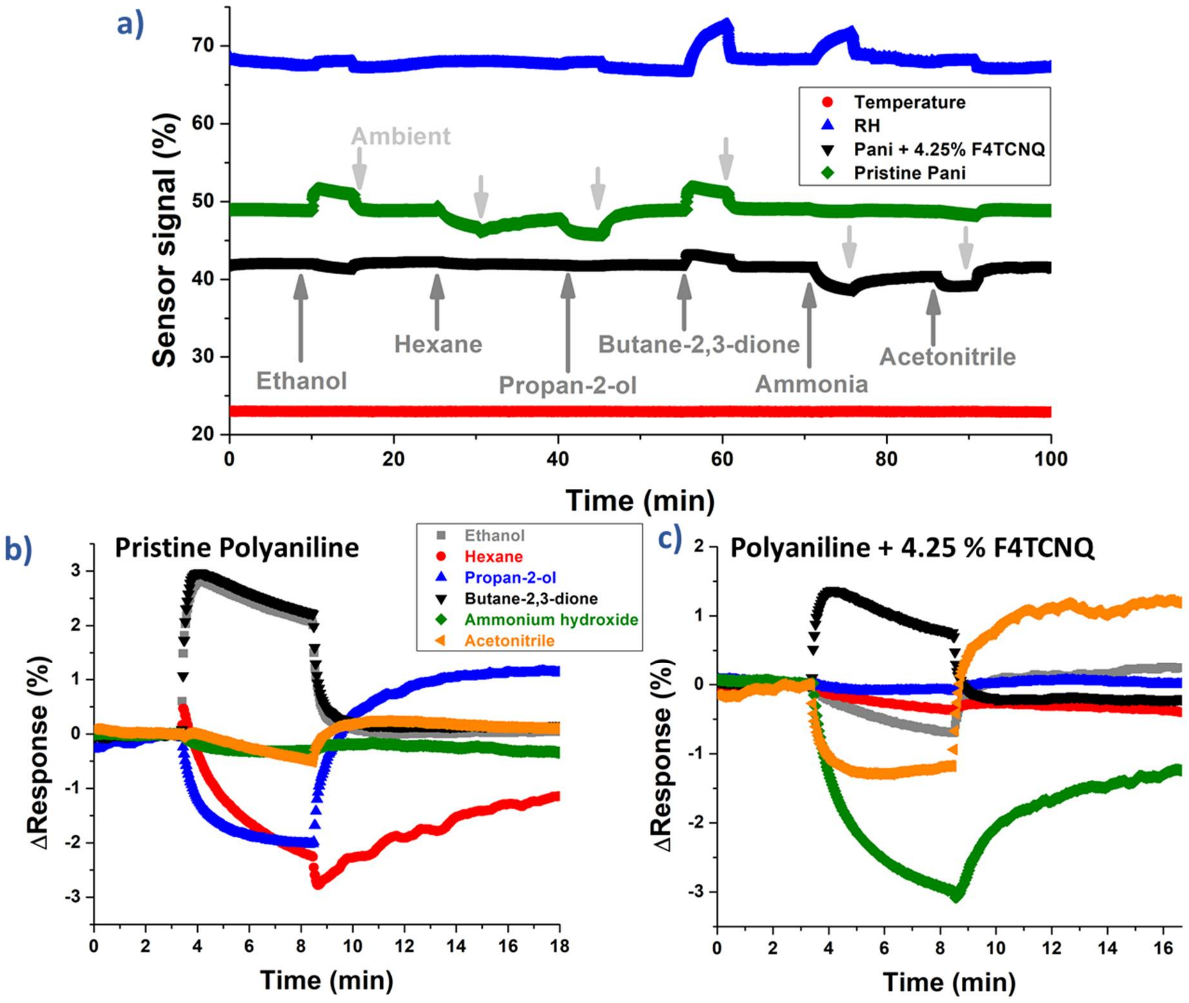


**Figure S5:** (**a**) Kinetic responses of a continuous measurement of the six analytes labeled by their injection time in grey. Light grey arrows mark the regeneration step in ambient conditions. (**b**) Overlapped view of the normalized sensor responses for a pristine polyaniline sensor. (**c**) Overlapped view of the normalized sensor responses of a polyaniline sensor doped with 4.25wt% F4TCNQ.

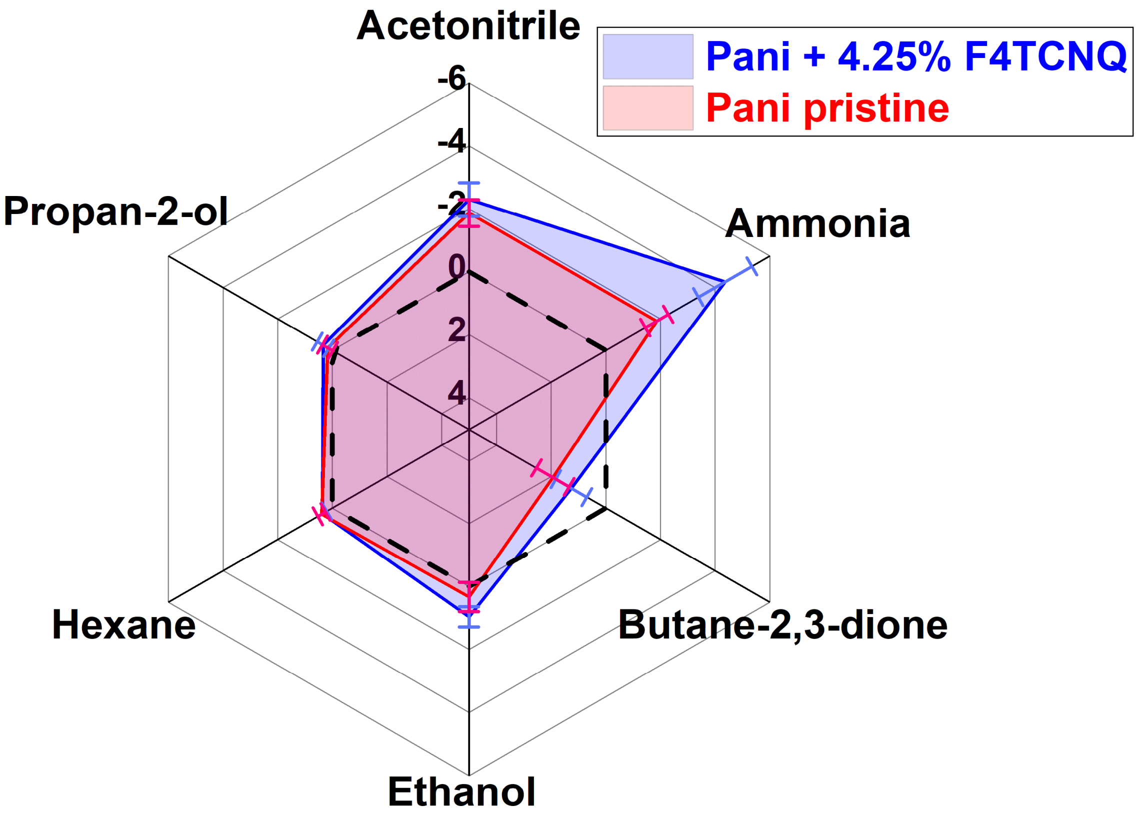


**Figure S6:** Radar plots for average responses and standard deviations to 6 analytes for pristine polyaniline (red) and polyaniline doped with 4.25wt% F4TCNQ (blue).
